# Light sedation strategies and posttraumatic stress disorder: a systematic review and network meta-analysis

**DOI:** 10.1186/cc14694

**Published:** 2015-09-28

**Authors:** Antonio Paulo Nassar Junior, Fernando G Zampieri, Otavio T Ranzani, Marcelo Park

**Affiliations:** 1 Hospital das Clinicas, Faculty of Medicine, University of São Paulo, SP, Brazil

## Introduction

Strategies aiming at light sedation are associated with decreased times on mechanical ventilation. However, awake or easily aroused patients may be prone to have greater prevalence of posttraumatic stress disorder. This systematic review and meta-analysis aimed to evaluate the safety of light sedation strategies in the prevalence of posttraumatic stress disorder.

## Methods

We searched MEDLINE, Scopus and Web of Science from inception to November 2014 for randomized controlled trials which compared a light sedation strategy with a deeper sedation strategy and addressed posttraumatic stress disorder prevalence as a specific outcome.

## Results

Five studies fulfilled our inclusion criteria and were included in the meta-analysis. Two studies compared daily sedation interruption with usual care (92 patients), two studies compared a light sedation protocol with daily sedation interruption (47 patients) and one study compared light and deep sedation (102 patients). Compared with usual sedation care/deep sedation, neither daily interruption of sedation (OR = 0.66, 95 % CI 0.22-1.98) nor a light sedation protocol (OR = 0.90, 95 % CI 0.27-3.05) were associated with increased risks on long-term PTSD prevalence. Heterogeneity was moderate (*I*^2 ^= 40 %).

## Conclusion

Light sedation strategies seem to be safe in terms of posttraumatic stress disorder prevalence. However, the small number of included trials and patients may not be sufficient to drive strong statements. Ongoing large trials will be able to answer this question.

